# Supplementary material for: A shared inflammatory signature across severe malaria syndromes manifested by transcriptomic, proteomic and metabolomic analyses
Source: Nat Commun. 2025 May 18;16:4620. doi: 10.1038/s41467-025-59281-5 (PMC12086225; doi:10.1038/s41467-025-59281-5)
Supplement: Supplementary file 1 — Supplementary Information [file 41467_2025_59281_MOESM1_ESM.pdf]

Supplementary Information

A shared inflammatory signature across severe malaria syndromes manifested by transcriptomic, proteomic and metabolomic analyses

Rafal S. Sobota[1,2], Emily M. Stucke[1], Drissa Coulibaly[3], Jonathan G. Lawton[1], Bryan E. Cummings[1], Savy Sebastian[1], Antoine Dara[3], James B. Munro[4], Amed Ouattara[1], Abdoulaye K. Kone[3], Bourama Kane[3], Karim Traoré[3], Bouréima Guindo[3], Bourama M. Tangara[3], Amadou Niangaly[3], Noah T. Ventimiglia[1], Modibo Daou[3], Issa Diarra[3], Youssouf Tolo[3], Mody Sissoko[3], Fayçal Maiga[3], Aichatou Diawara[3], Amidou Traore[3], Ali Thera[3], Matthew B. Laurens[1], Kirsten E. Lyke[1], Bourema Kouriba[3], Ogobara K. Doumbo[3], Christopher V. Plowe[1], David R. Goodlett[5], Joana C. Silva[4], Mahamadou A. Thera[3], Mark A. Travassos[1]

Corresponding author: Mark A. Travassos, MD, MSc, University of Maryland School of Medicine, 685 West Baltimore Street, Room 480, Baltimore, MD 21201, mtravass@som.umaryland.edu, (410) 706-8695.

Affiliations:

[1]Malaria Research Program, Center for Vaccine Development and Global Health, University of Maryland School of Medicine; Baltimore, Maryland, United States of America.

[2]Ken and Ruth Davee Department of Neurology, Northwestern University; Chicago, Illinois, United States of America.

[3]Malaria Research and Training Center, University of Sciences, Techniques and Technologies; Bamako, Mali.

[4]Institute for Genome Sciences, University of Maryland School of Medicine, Baltimore, MD, USA

[5]Department of Biochemistry and Microbiology, University of Victoria; Victoria, BC, Canada.

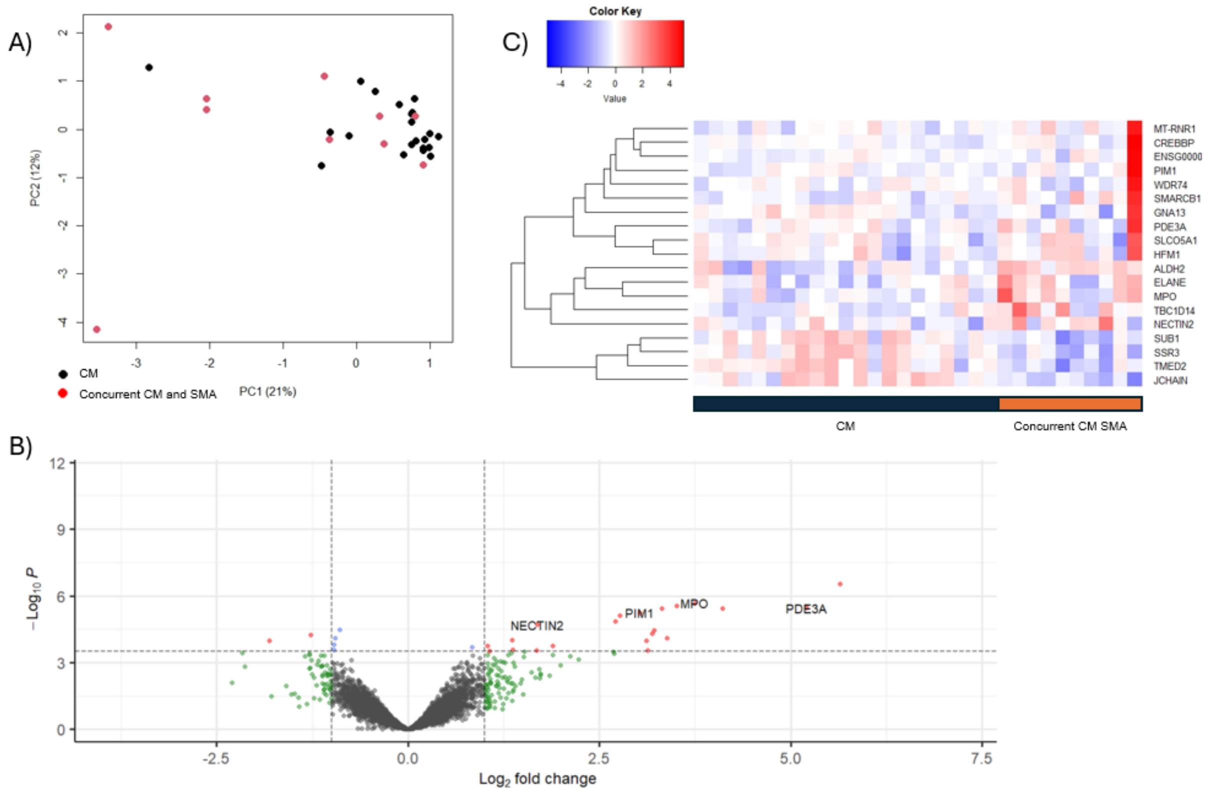

Supplemental Figure 1. Differential gene expression analysis comparing cerebral malaria to concurrent cerebral malaria and severe malarial anemia. A) Principal component analysis showing the separation of CM from concurrent CM and SMA along the first two principal components. B) Volcano plot showing the significance of association from a quasi-likelihood F test based on the negative binomial distribution (red and blue FDR adjusted p-value < 0.05) and effect size (green and red > 1 absolute $\log_2$-fold change). C) Heatmap of differentially expressed transcripts showing the clustering of associating genes from a quasi-likelihood F test (y-axis).

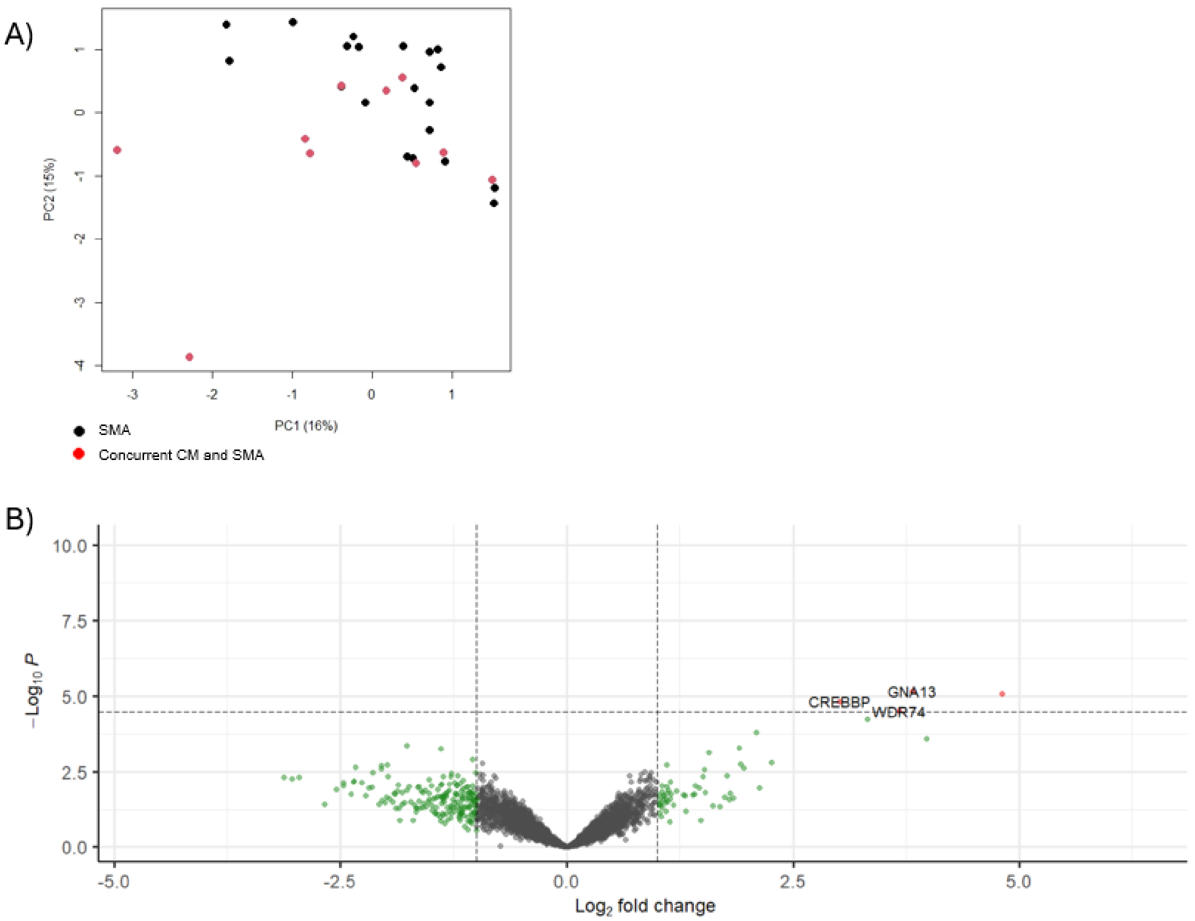

Supplemental Figure 2. Differential gene expression analysis comparing severe malarial anemia to concurrent cerebral malaria and severe malarial anemia. A) Principal component analysis showing the separation of SMA from concurrent CM and SMA along the first two principal components. B) Volcano plot showing significance of association from a quasi-likelihood F test based on the negative binomial distribution (red FDR adjusted p-value < 0.05) and effect size (green and red > 1 absolute log_2-fold change).

A)

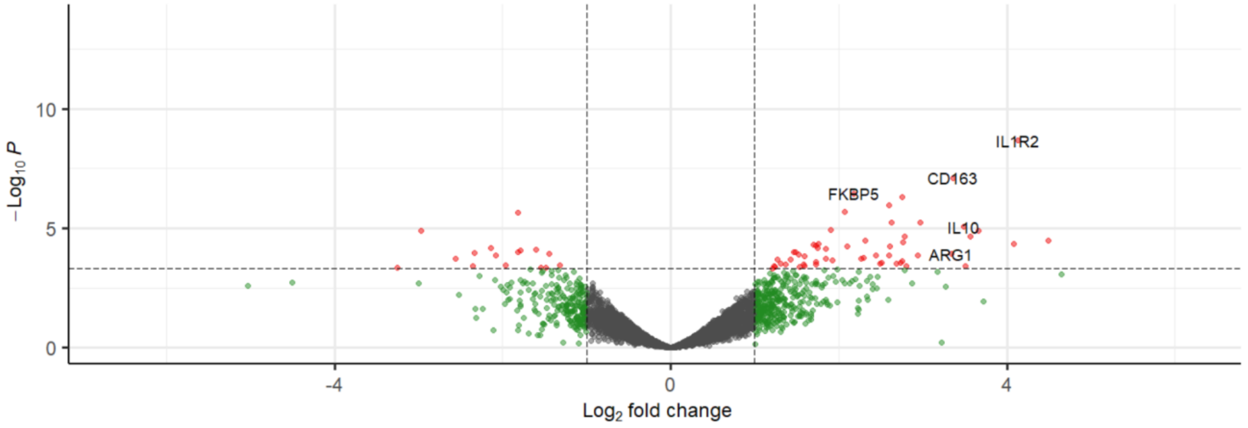

B)

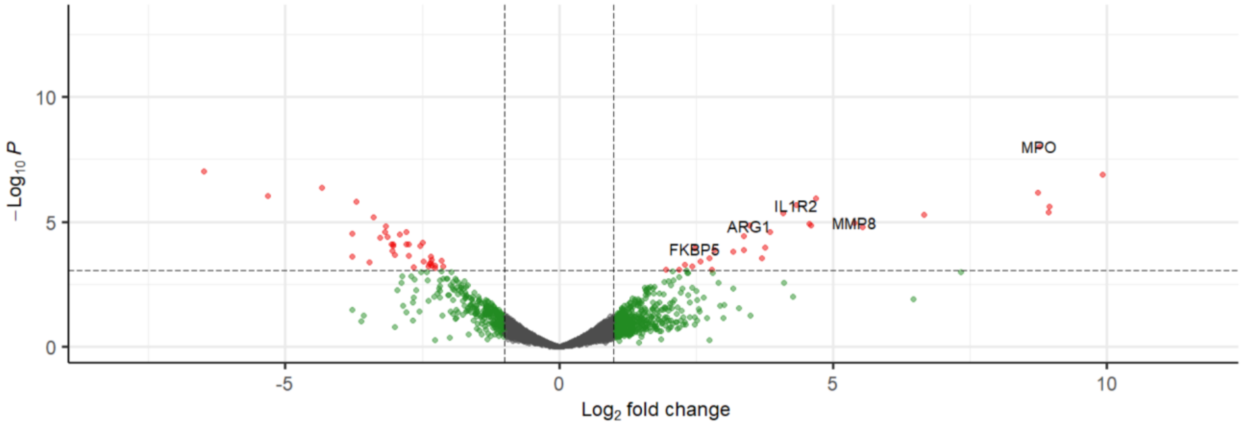

Supplemental Figure 3. Volcano plots showing the significance of association assessed with a two-tailed paired t test (red FDR adjusted p-value < 0.05) and effect size (green and red > 1 absolute $\log_2$-fold change) in transcriptome comparisons of A) cerebral malaria to uncomplicated malaria controls with a history of cerebral malaria and B) concurrent cerebral malaria and severe malarial anemia to uncomplicated malaria controls with a history of cerebral malaria.

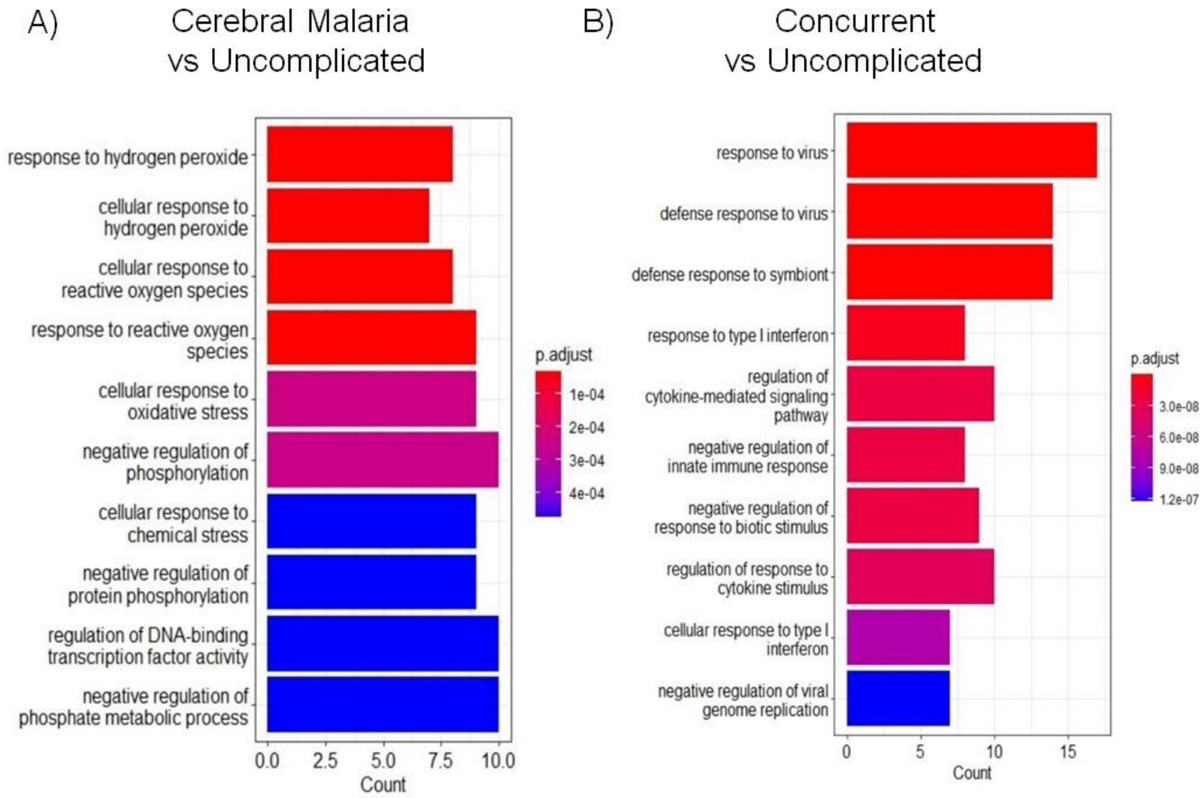

Supplemental Figure 4. Bar plots of gene counts in the top 10 gene ontology biological processes associating in transcriptome comparisons. Enrichment analysis was performed using the clusterProfiler package in R. Enriched terms were determined using a hypergeometric test, with p values <0.05 considered significant. Pathway names are represented on the Y-axis, and gene counts on the X-axis. Color spectrum represents the extent of statistical association. A) cerebral malaria, and B) concurrent cerebral malaria and severe malarial anemia, are compared to uncomplicated malaria controls with a history of cerebral malaria

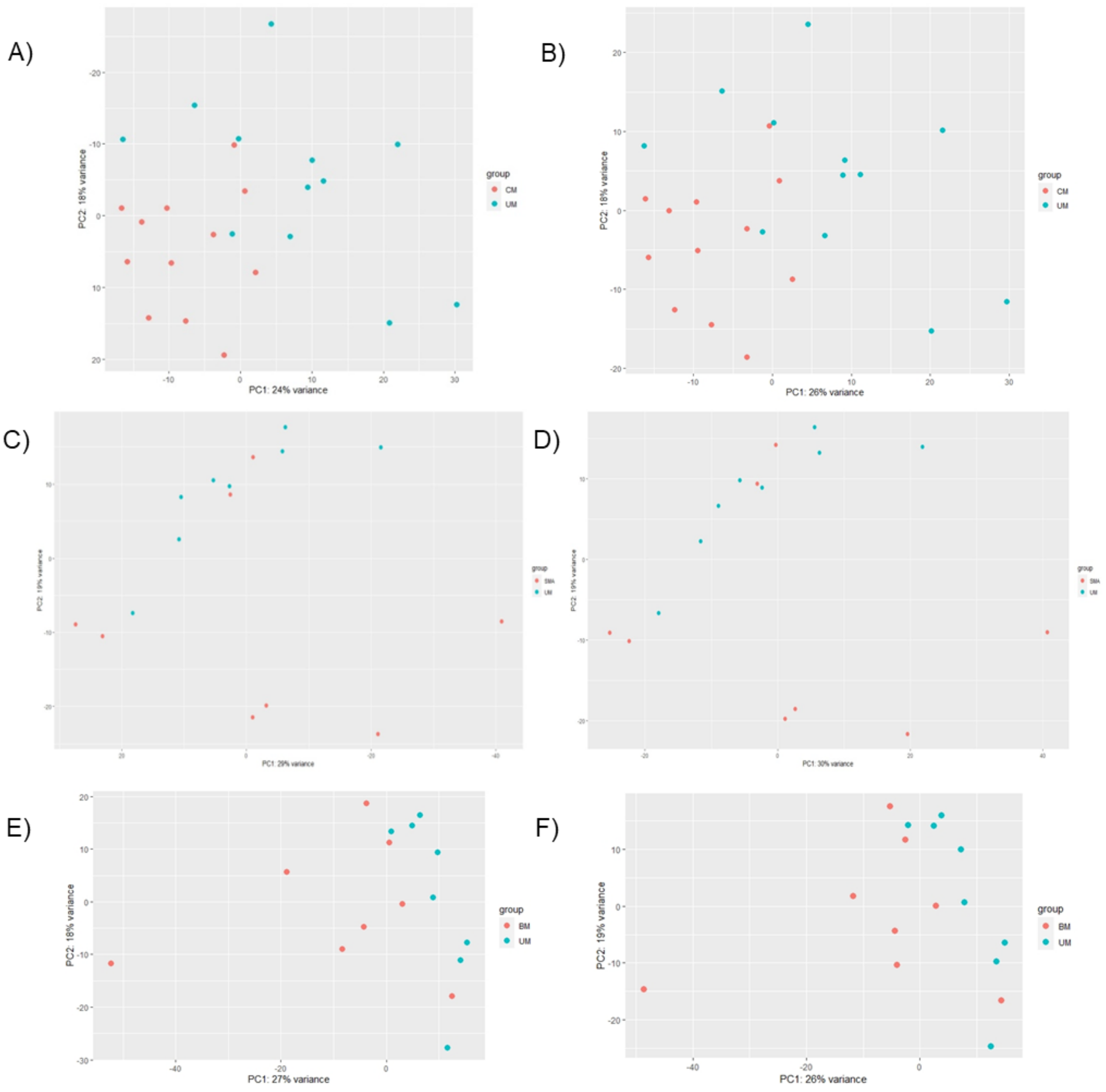

Supplemental Figure 5. Principle component analyses of gene expression counts with and without adjustment for cell proportions. A) Cerebral malaria versus controls without a history of CM, unadjusted. B) Cerebral malaria versus controls without a history of CM, adjusted for B cell, T cell, and neutrophil proportions. C) Severe malarial anemia versus controls without a history of CM, unadjusted. D) Severe malarial anemia versus controls without a history of CM, adjusted for B cell, T cell, and neutrophil proportions. E) Concurrent cerebral malaria and severe malarial anemia (BM) versus controls without a history of CM (UM), unadjusted. F) Concurrent cerebral malaria and severe malarial anemia (BM) versus controls without a history of CM (UM), adjusted for B cell, T cell, and neutrophil proportions

Supplemental Table 1. Top 20 transcripts with the most significant association from differential gene expression analyses between CM in comparison to controls without a history of CM. Significance of association was assessed with a quasi-likelihood F test based on the negative binomial distribution. False discovery rate was used to correct for multiple testing.

Gene	logFC	logCPM	p value	FDR
IL1R2	4.41	8.73	2.09E-15	1.75E-11
FKBP5	2.58	7.72	4.76E-13	2.00E-09
MMP8	4.20	5.11	9.75E-12	2.73E-08
CD163	4.13	5.49	2.23E-11	4.68E-08
MMP9	2.04	7.79	6.13E-10	1.03E-06
OLFM4	4.48	4.32	5.55E-09	7.77E-06
IL1R1	2.11	5.81	1.37E-08	1.64E-05
IL18R1	2.61	6.35	2.11E-08	2.22E-05
IRAK3	1.66	8.01	3.74E-08	3.42E-05
ADARB1	-1.60	7.17	4.07E-08	3.42E-05
SPATC1	2.99	4.50	5.43E-08	4.15E-05
SAMSN1	2.35	6.29	4.16E-07	2.68E-04
ZDHHC20	1.62	6.01	4.46E-07	2.68E-04
RBM33	-1.38	8.29	4.47E-07	2.68E-04
TNFRSF25	-1.37	6.49	5.38E-07	3.01E-04
FGD4	1.60	5.87	6.42E-07	3.05E-04
ENSG00000254420	2.69	4.76	6.56E-07	3.05E-04
KCNE1	2.42	7.43	6.85E-07	3.05E-04
TRIB1	2.15	4.70	6.89E-07	3.05E-04
SLCO4A1	2.41	4.48	8.40E-07	3.53E-04

Supplemental Table 2. Gene Ontology biological processes with the strongest associations when comparing gene expression between CM versus uncomplicated controls without a history of CM. Statistical significance was assessed using a one-sided hypergeometric test. P values were adjusted for multiple testing using the Benjamini-Hochberg method.

Term for biological process	Gene Ratio	Adjusted p value
T cell activation	33/365	2.19E-06
Positive regulation of cytokine production	31/365	4.16E-06
Regulation of immune effector process	26/365	4.16E-06
Leukocyte mediated immunity	29/365	9.89E-06
Immune response-regulating signaling pathway	30/365	9.89E-06
Regulation of T cell activation	24/365	2.08E-05
Adaptive immune response based on somatic recombination of immune receptors built from Immunoglobulin superfamily domains	25/365	2.08E-05
Negative regulation of immune system process	27/365	6.23E-05
Lipopolysaccharide-mediated signaling pathway	10/365	8.82E-05
Negative regulation of immune effector process	13/365	8.82E-05

Supplemental Table 3. Upregulated KEGG pathways significantly associated with transcription in CM compared to controls with uncomplicated disease without history of CM. Statistical significance was assessed using a one-sided hypergeometric test.

Pathway	total	upregulated	p value
Chemical carcinogenesis - reactive oxygen species	110	9	4.01E-03
Regulation of actin cytoskeleton	91	7	0.015
Focal adhesion	92	7	0.016
Calcium signaling pathway	117	8	0.018
Human papillomavirus infection	145	9	0.022
Other types of O-glycan biosynthesis	9	2	0.025
Arrhythmogenic right ventricular cardiomyopathy	41	4	0.028
Circadian rhythm	12	2	0.044

Supplemental Table 4. Transcripts significant association from differential gene expression analyses between SMA in comparison to controls without a history of CM. Significance of association was assessed with a quasi-likelihood F test based on the negative binomial distribution, and false discovery rate was used to correct for multiple testing.

Gene	logFC	logCPM	p value	FDR
DAAM2	4.83	4.37	1.88E-07	1.54E-03
LTF	3.87	6.59	5.74E-07	2.36E-03
ARG1	2.82	6.24	1.81E-06	4.97E-03
THSD4	4.35	5.33	4.41E-06	8.95E-03
PRTN3	6.01	3.98	6.06E-06	8.95E-03
AZU1	4.62	4.37	6.53E-06	8.95E-03
LCN2	3.52	5.63	8.20E-06	9.63E-03
IL1R2	3.02	8.85	1.39E-05	0.014
ZBTB16	2.72	4.75	1.59E-05	0.014
TPST1	2.96	5.20	2.82E-05	0.021
PRKG1	3.94	5.86	2.95E-05	0.021
MMP8	2.96	5.07	3.37E-05	0.021
IRS2	1.91	5.65	3.49E-05	0.021
TMEM204	-2.18	4.79	3.52E-05	0.021
FKBP5	1.74	8.03	5.31E-05	0.028
SPTA1	5.00	3.61	5.53E-05	0.028
CHN2	2.05	5.29	6.44E-05	0.031
MYC	-1.63	6.26	7.66E-05	0.035
OAS3	-2.02	7.04	1.04E-04	0.045

Supplemental Table 5. Gene Ontology biological processes with the strongest associations when comparing gene expression between SMA versus uncomplicated controls without a history of CM. Statistical significance was assessed using a one-sided hypergeometric test. P values were adjusted for multiple testing using the Benjamini-Hochberg method.

Term for biological process	Gene Ratio	Adjusted p value
Negative regulation of cytokine production	5/19	6.56E-03
Tumor necrosis factor production	4/19	6.56E-03
Regulation of tumor necrosis factor production	4/19	6.56E-03
Tumor necrosis factor superfamily cytokine production	4/19	6.56E-03
Regulation of tumor necrosis factor superfamily cytokine production	4/19	6.56E-03
Killing of cells of other organism	3/19	7.16E-03
Neutrophil-mediated killing of symbiont cell	2/19	8.08E-03
Negative regulation of cytokine-mediated signaling pathway	3/19	8.08E-03
Iron ion homeostasis	3/19	8.08E-03
Negative regulation of response to cytokine stimulus	3/19	8.08E-03

Supplemental Table 6. Upregulated KEGG pathways significantly associated with transcription in SMA compared to controls with uncomplicated disease without history of CM. Statistical significance was assessed using a one-sided hypergeometric test.

Pathway	total	upregulated	p value
Vitamin B6 metabolism	2	1	3.89E-03
Valine, leucine and isoleucine biosynthesis	3	1	5.83E-03
Nicotinate and nicotinamide metabolism	4	1	7.77E-03
Metabolic pathways	498	4	0.014
Glycine, serine and threonine metabolism	16	1	0.031
Arachidonic acid metabolism	16	1	0.031
Tryptophan metabolism	19	1	0.036
Retinol metabolism	20	1	0.038
Cysteine and methionine metabolism	21	1	0.040
Valine, leucine and isoleucine degradation	22	1	0.042
Tyrosine metabolism	22	1	0.042
Inositol phosphate metabolism	26	1	0.049

Supplemental Table 7. Top 20 transcripts with the most significant association from differential gene expression analyses between concurrent CM and SMA in comparison to controls without a history of CM. Significance of association was assessed with a quasi-likelihood F test based on the negative binomial distribution. False discovery rate was used to correct for multiple testing.

Gene	logFC	logCPM	p value	FDR
OLFM4	6.90	5.77	2.79E-09	1.75E-05
XAF1	-2.39	7.44	1.95E-06	2.77E-03
IL1R2	4.04	8.71	2.04E-06	2.77E-03
DEFA1	5.01	5.27	2.11E-06	2.77E-03
PER1	2.88	5.50	2.44E-06	2.77E-03
IL1R1	3.07	6.29	2.65E-06	2.77E-03
PRTN3	6.28	5.50	4.88E-06	4.14E-03
MMP9	2.84	8.70	5.29E-06	4.14E-03
PFKFB2	2.87	5.10	5.99E-06	4.17E-03
GYG1	2.05	6.57	8.10E-06	5.08E-03
AATBC	-3.11	5.74	9.10E-06	5.19E-03
FKBP5	2.21	7.66	1.19E-05	6.24E-03
EDIL3	6.44	5.82	1.31E-05	6.31E-03
ZDHHC20	2.04	6.06	1.61E-05	7.19E-03
ISG15	-2.23	5.95	2.11E-05	8.80E-03
ZBP1	-1.86	6.69	2.27E-05	8.90E-03
OAS3	-2.33	6.38	2.80E-05	0.010
MX1	-1.81	6.84	3.48E-05	0.012
ST6GALNAC3	2.84	4.87	3.81E-05	0.013
RBFOX1	6.14	5.47	4.47E-05	0.013

Supplemental Table 8. Gene Ontology biological processes with the strongest associations when comparing gene expression between concurrent CM and SMA versus uncomplicated controls without a history of CM. Statistical significance was assessed using a one-sided hypergeometric test. P values were adjusted for multiple testing using the Benjamini-Hochberg method.

Term for biological process	Gene Ratio	Adjusted p value
Response to virus	14/63	2.35E-08
Defense response to virus	11/63	6.16E-07
Defense response to symbiont	11/63	6.16E-07
Negative regulation of immune response	9/63	6.47E-06
Regulation of cytokine-mediated signaling pathway	8/63	1.04E-05
Response to type I interferon	6/63	1.04E-05
Cytokine-mediated signaling pathway	12/63	1.05E-05
Regulation of response to cytokine stimulus	8/63	1.32E-05
Negative regulation of innate immune response	6/63	2.36E-05
Positive regulation of interferon-beta production	5/63	2.99E-05

Supplemental Table 9. KEGG pathways significantly associated with transcription in concurrent CM and SMA compared to controls with uncomplicated disease without history of CM. Statistical significance was assessed using a one-sided hypergeometric test.

A. Upregulated pathways in the comparison of concurrent CM and SMA cases to controls without a history of CM

Pathway	total	upregulated	p value
Pancreatic secretion	40	2	0.028
Ubiquitin mediated proteolysis	42	2	0.030
Vitamin digestion and absorption	7	1	0.045

B. Downregulated pathway in the comparison of concurrent CM and SMA cases to controls without a history of CM

Pathway	total	downregulated	p value
Necroptosis	52	2	0.019

Supplemental Table 10. Demographic data for controls with and without a history of CM. * denotes mean (standard deviation). Statistical significance assessed using a two-tailed t-test.

	Controls w/ hx of SM	Controls w/o hx of SM	p value
n	16	16	
Female	8	8	
Age* (yrs)	3.39 (1.04)	3.33 (0.97)	0.82
Parasitemia* (parasite/μL)	37764 (63118)	22076 (40138)	0.42
BCS			
5	16	16	
Hgb* (g/dL)	9.34 (1.56)	9.19 (1.19)	0.80

Supplemental Table 11. Top 20 significantly associated transcripts expressed at a higher level in SMA than CM. Significance of association was assessed with a quasi-likelihood F test based on the negative binomial distribution. False discovery rate was used to correct for multiple testing.

Gene	logFC	logCPM	p value	FDR
H1-2	1.86	6.69	6.52E-08	4.15E-04
H1-0	2.37	6.25	9.97E-07	1.55E-03
SLC2A1	2.96	7.85	1.35E-06	1.55E-03
RHCE	3.33	6.23	1.15E-06	1.55E-03
RHD	4.10	6.64	1.24E-06	1.55E-03
SLC6A19	5.28	7.93	1.46E-06	1.55E-03
ACP5	2.11	6.68	1.88E-06	1.71E-03
CARHSP1	1.06	6.33	3.84E-06	2.67E-03
CCS	1.31	6.19	4.61E-06	2.67E-03
MED25	1.43	6.29	5.46E-06	2.67E-03
MAP3K20	1.77	5.62	5.43E-06	2.67E-03
HMBS	2.17	5.91	4.20E-06	2.67E-03
BSG	2.30	10.47	5.11E-06	2.67E-03
NME4	1.08	5.22	9.51E-06	4.17E-03
ANK1	2.63	7.24	9.80E-06	4.17E-03
FIS1	1.56	7.40	1.18E-05	4.70E-03
MYO7B	1.12	4.31	1.95E-05	6.06E-03
UROD	1.41	5.59	1.96E-05	6.06E-03
SLC1A5	2.38	7.18	1.69E-05	6.06E-03
BMP2K	2.51	7.28	2.09E-05	6.06E-03

Supplemental Table 12. Top 20 significantly associated transcripts expressed at a higher level in CM than SMA. Significance of association was assessed with a quasi-likelihood F test based on the negative binomial distribution. False discovery rate was used to correct for multiple testing.

Gene	logFC	logCPM	p value	FDR
GBP3	-1.26	4.86	3.10E-05	8.06E-03
H2BC21	-1.31	4.90	3.61E-05	8.22E-03
SELENOT	-0.79	5.97	5.35E-05	9.47E-03
ATP8B2	-0.93	5.12	8.32E-05	0.013
EDEM1	-0.91	5.39	2.14E-04	0.020
CCDC93	-0.94	4.94	2.58E-04	0.023
IL7R	-0.82	7.02	2.92E-04	0.025
TMEM123	-0.75	6.70	3.02E-04	0.025
TRIM25	-1.00	7.80	3.68E-04	0.028
ZNF101	-0.73	5.20	4.03E-04	0.029
PPT1	-0.71	6.58	4.41E-04	0.031
TMEM273	-1.10	4.30	4.91E-04	0.033
CNIH1	-0.97	4.40	4.96E-04	0.033
TAGAP	-0.58	6.58	6.36E-04	0.038
TMEM126B	-0.96	4.00	6.93E-04	0.039
CD164	-0.69	6.98	6.80E-04	0.039
CACYBP	-0.95	5.71	7.30E-04	0.040
PATL1	-0.84	4.93	7.77E-04	0.041
HACD4	-0.92	5.25	8.03E-04	0.041
MORF4L2	-0.78	4.93	8.49E-04	0.042

Supplemental Table 13. Gene Ontology biological processes with the strongest associations when comparing gene expression between CM versus SMA. Statistical significance was assessed using a one-sided hypergeometric test. P values were adjusted for multiple testing using the Benjamini-Hochberg method. This table corresponds to Figure 4D.

Term for biological process	Gene Ratio	Adjusted p value
Cellular response to toxic substance	10/159	2.22E-04
Cellular oxidant detoxification	9/159	2.22E-04
Cellular detoxification	9/159	4.86E-04
Protoporphyrinogen IX metabolic process	4/159	8.94E-04
Detoxification	9/159	2.62E-03
Hydrogen peroxide catabolic process	5/159	2.62E-03
Response to toxic substance	11/159	4.92E-03
Proteasomal protein catabolic process	15/159	5.45E-03
Porphyrin-containing compound metabolic process	5/159	0.015
Erythrocyte differentiation	7/159	0.017

Supplemental Table 14. KEGG pathways significantly associated with transcription in comparisons between severe malaria subtypes. Statistical significance was assessed using a one-sided hypergeometric test.

A. Upregulated pathways in SMA compared to CM

Pathway	total	upregulated	p value
GABAergic synapse	38	4	8.56E-03
Taste transduction	22	3	0.011
Cortisol synthesis and secretion	33	3	0.033
Bile secretion	34	3	0.036
Endocytosis	59	4	0.037
Morphine addiction	36	3	0.041
Pancreatic secretion	38	3	0.047
Oxytocin signaling pathway	64	4	0.048

B. Upregulated pathways in CM compared to SMA

Pathway	total	upregulated	p value
Taurine and hypotaurine metabolism	4	1	0.017
Citrate cycle (TCA cycle)	12	1	0.052

Supplemental Table 15. Top 20 significantly associated transcripts in differential expression analysis of CM versus concurrent CM and SMA. Significance of association was assessed with a quasi-likelihood F test based on the negative binomial distribution. False discovery rate was used to correct for multiple testing.

Gene	logFC	logCPM	p value	FDR
ENSG00000258486	5.64	11.17	2.97E-07	2.03E-03
MPO	3.75	6.68	2.10E-06	4.21E-03
SMARCB1	3.51	8.69	2.77E-06	4.21E-03
PDE3A	5.20	8.82	3.52E-06	4.21E-03
CREBBP	3.32	8.76	3.62E-06	4.21E-03
WDR74	4.11	7.09	3.70E-06	4.21E-03
PIM1	3.03	9.57	5.81E-06	5.66E-03
TBC1D14	2.77	9.24	7.71E-06	6.58E-03
MT-RNR1	2.71	9.93	1.37E-05	0.010
NECTIN2	1.69	5.45	1.95E-05	0.013
SUB1	-0.89	7.10	3.24E-05	0.020
ELANE	3.22	5.81	3.45E-05	0.020
HFM1	3.19	12.47	4.91E-05	0.026
SSR3	-1.27	5.94	5.69E-05	0.028
SLCO5A1	3.39	10.04	7.69E-05	0.034
TMED2	-0.95	6.19	8.06E-05	0.034
ALDH2	1.36	5.10	9.58E-05	0.038
GNA13	3.11	7.43	0.000103	0.038
JCHAIN	-1.82	9.77	0.000105	0.038

Supplemental Table 16. KEGG pathways significantly associated with transcription in comparisons between severe malaria subtypes. Statistical significance was assessed using a one-sided hypergeometric test.

A. Upregulated pathways in concurrent CM and SMA compared to CM alone

Pathway	total	upregulated	p value
TGF-beta signaling pathway	36	2	2.72E-03
Signaling pathways regulating pluripotency of stem cells	49	2	5.00E-03
Apelin signaling pathway	50	2	5.20E-03
Steroid biosynthesis	9	1	0.020
Cytokine-cytokine receptor interaction	103	2	0.021
Porphyrin metabolism	13	1	0.028
Olfactory transduction	19	1	0.041
Mineral absorption	23	1	0.049

B. Upregulated pathways in CM compared to concurrent CM and SMA

Pathway	total	upregulated	p value
Vibrio cholerae infection	14	1	8.19E-03
Pancreatic secretion	40	1	0.023
GnRH signaling pathway	42	1	0.024
Salivary secretion	45	1	0.026
TNF signaling pathway	48	1	0.028
Parathyroid hormone synthesis, secretion and action	54	1	0.031

Supplemental Table 17. Significantly associated transcripts in differential expression analysis of SMA versus concurrent CM and SMA. Significance of association was assessed with a quasi-likelihood F test based on the negative binomial distribution.

Gene	logFC	logCPM	p value	FDR
GNA13	3.82	7.12	6.92E-06	0.025
ENSG00000258486	4.81	11.00	8.28E-06	0.025
CREBBP	3.02	8.61	1.51E-05	0.031
WDR74	3.67	7.01	3.16E-05	0.049

Supplemental Table 18. Upregulated KEGG pathways in comparing concurrent CM and SMA compared to SMA alone. Statistical significance was assessed using a one-sided hypergeometric test.

Pathway	total	upregulated	p value
Steroid biosynthesis	8	1	5.19E-03
TGF-beta signaling pathway	35	1	0.023
Morphine addiction	35	1	0.023
Signaling pathways regulating pluripotency of stem cells	45	1	0.029
Estrogen signaling pathway	60	1	0.038

Supplemental Table 19. Demographic data for the study participants in proteomic analyses for CM versus controls without a history of CM. * denotes mean (standard deviation). Statistical significance assessed using a two-tailed t-test.

	Cases	Controls	p value
n	14	14	
Female	8	8	
Age* (yrs)	2.56 (1.22)	2.93 (1.12)	0.45
Parasitemia* (parasite/μL)	107330 (132947)	28670 (46454)	0.039
BCS			
5	0	14	
2	11	0	
1	3	0	
Hgb* (g/dL)	8.06 (2.02)	8.62 (1.82)	0.36

Supplemental Table 20. Protein upregulated in CM versus controls without a history of CM. Statistical significance assessed with a two-tailed paired t test, using an alpha level of 0.05.

Gene	p value	LogFC
TIMP1	1.71E-03	1.80
CD14	2.00E-03	0.64
CAT	2.70E-03	1.83
LBP	3.62E-03	1.25
ACTBL2	3.70E-03	1.75
HSP90B1	5.23E-03	0.70
SERPINF1	5.70E-03	0.38
VCAM1	6.15E-03	0.65
VASN	6.49E-03	0.73
SERPINA3	7.60E-03	1.08
CPN1	0.010	0.50
ICAM1	0.014	1.61
ACTA1	0.016	1.76
ACTB	0.017	2.19
LCP1	0.017	1.05
SERPINA10	0.019	0.70
LILRA3	0.019	0.53
ENPP2	0.019	0.80
PRG4	0.024	0.83
A1BG	0.025	0.22
LGALS3BP	0.026	0.57
LRG1	0.035	0.55
FCN2	0.037	1.01
F9	0.038	0.70
GC	0.042	0.26
VWF	0.046	0.74
ITIH3	0.046	0.49
SERPING1	0.047	0.44

Supplemental Table 21. Protein downregulated in CM versus controls without a history of CM. Statistical significance assessed with a two-tailed paired t test, using an alpha level of 0.05.

Gene	p value	LogFC
SERPIND1	1.65E-03	-0.55
APOM	1.99E-03	-1.03
PLG	3.26E-03	-0.23
THBS1	4.67E-03	-1.47
PPBP	5.55E-03	-1.13
C3	9.24E-03	-0.47
PF4	0.011	-1.01
CFP	0.013	-0.78
C1QB	0.020	-0.55
TTR	0.020	-0.43
GSN	0.027	-0.37
MBL2	0.039	-0.95
LUM	0.039	-0.49
HRG	0.045	-0.28
SHBG	0.048	-0.48

Supplemental Table 22. Gene Ontology biological processes of protein upregulated in CM versus controls with uncomplicated malaria and no history of CM. Statistical significance was assessed using a one-sided hypergeometric test.

Term for biological process	total	upregulated	p value
response to abiotic stimulus	15	9	4.95E-05
response to endogenous stimulus	26	11	3.88E-04
response to hypoxia	6	5	3.94E-04
response to decreased oxygen levels	6	5	3.94E-04
response to oxygen levels	6	5	3.94E-04
response to radiation	4	4	5.30E-04
response to organonitrogen compound	16	8	8.31E-04
response to nitrogen compound	16	8	8.31E-04
cellular response to organic substance	33	12	1.06E-03
cellular response to endogenous stimulus	18	8	2.24E-03
Aging	11	6	2.55E-03
response to organic substance	49	14	5.65E-03
cellular response to organonitrogen compound	9	5	5.79E-03
response to peptide	9	5	5.79E-03
cellular response to nitrogen compound	9	5	5.79E-03
response to ethanol	6	4	6.27E-03
response to organic cyclic compound	17	7	7.78E-03
cellular response to oxygen-containing compound	17	7	7.78E-03

Supplemental Table 23. Gene Ontology biological processes of protein downregulated in CM versus controls with uncomplicated malaria and no history of CM. Statistical significance was assessed using a one-sided hypergeometric test.

Term for biological process	total	downregulated	p value
Chemotaxis	11	5	5.30E-04
Taxis	11	5	5.30E-04
cellular component disassembly	8	4	1.50E-03
cell chemotaxis	8	4	1.50E-03
antimicrobial humoral immune response mediated by antimicrobial peptide	4	3	1.55E-03
granulocyte chemotaxis	5	3	3.70E-03
granulocyte migration	5	3	3.70E-03
carbohydrate transport	2	2	5.91E-03
hexose transmembrane transport	2	2	5.91E-03
monosaccharide transmembrane transport	2	2	5.91E-03
transforming growth factor beta1 production	2	2	5.91E-03
regulation of transforming growth factor beta1 production	2	2	5.91E-03
positive regulation of transforming growth factor beta1 production	2	2	5.91E-03
carbohydrate transmembrane transport	2	2	5.91E-03
endothelial cell chemotaxis	2	2	5.91E-03
regulation of cysteine-type endopeptidase activity involved in apoptotic process	2	2	5.91E-03
chemokine-mediated signaling pathway	2	2	5.91E-03
positive regulation of epithelial cell apoptotic process	2	2	5.91E-03
glucose transmembrane transport	2	2	5.91E-03
response to chemokine	2	2	5.91E-03
cellular response to chemokine	2	2	5.91E-03
regulation of cysteine-type endopeptidase activity	2	2	5.91E-03
regulation of endothelial cell chemotaxis	2	2	5.91E-03
negative regulation of endothelial cell chemotaxis	2	2	5.91E-03

Supplemental Table 24. Top 20 serum metabolites upregulated in CM versus uncomplicated malaria without history of CM. Statistical significance was assessed with a two-tailed paired t test, using an alpha level of 0.05, and false discovery rate was used for multiple testing adjustment.

Name	LogFC	Adj p value
5-hydroxy-2,2,6,6-tetramethyl-4-{2-methyl-1-[2,4,6-trihydroxy-3-(2-methylpropanoyl)phenyl]propyl}cyclohex-4-ene-1,3-dione	4.03	9.86E-04
3-Hydroxybutyric acid	2.54	1.42E-03
Estradiol-17beta-glucuronide	3.91	3.07E-03
Cortisol	1.59	3.61E-03
Prostaglandin A2	3.43	4.17E-03
Ethopabate	3.88	4.61E-03
Formiminoglutamic Acid	4.64	4.61E-03
3-(3,4-Dihydroxyphenyl)-N-(3-oxopropyl)propanamide	2.38	4.76E-03
17(S)-HpDHA	3.20	4.92E-03
Glyceraldehyde	0.48	5.25E-03
D-Phenylalanine	0.94	5.25E-03
beta-D-Glucopyranuronic acid	2.09	5.25E-03
trans-Cinnamic acid	1.14	5.29E-03
Taurochenodeoxycholic acid	2.92	5.37E-03
alpha-Aminoadipic acid	2.24	5.78E-03
N-{3-[(4-Acetamidobutyl)amino]propyl}acetamide	2.16	6.09E-03
D-Ribofuranosylcreatine	2.86	6.09E-03
Paracetamol	6.24	6.47E-03
3-(4-Methyl-3-pentenyl)thiophene	1.04	6.50E-03
Levulinic acid	1.48	7.72E-03

Supplemental Table 25. Serum metabolites downregulated in CM versus uncomplicated malaria without history of CM. Statistical significance was assessed with a two-tailed paired t test, using an alpha level of 0.05, and false discovery rate was used for multiple testing adjustment.

Name	LogFC	Adj p value
6-[(6-Aminohexanoyl)amino]hexanoic acid	-2.99	7.96E-03
Linoleic Acid	-0.79	0.017
Tetrahydro-2-furanylmethyl hydrogen sulfate	-0.5	0.021
phe-ile	-2.22	0.025
(2E,2'E)-N,N'-1,4-Butanediylbis[3-(3,4-dihydroxyphenyl)acrylamide]	-0.6	0.026
L-(+)-Arginine	-1.04	0.038
Oleic Acid	-0.57	0.045
Allopurinol	-3.27	0.049

Supplemental Table 26. Pathway comparison of metabolites upregulated in CM compared to controls with uncomplicated malaria and no history of CM using MetaboAnalyst 5.0. Enrichment analyses interrogated the chemical structure sub-class category and pathway analyses were performed using the KEGG *Homo sapiens* pathway library and a two-tailed hypergeometric test.

	Total	Expected	Hits	p	Impact
Phenylalanine, tyrosine, and tryptophan biosynthesis	4	0.12	2	4.77E-03	1
Phenylalanine metabolism	10	0.29	2	3.20E-02	0.36
Lysine degradation	25	0.73	3	3.37E-02	0.14
Alanine, aspartate, and glutamate metabolism	28	0.81	3	4.51E-02	0.05

Supplemental Table 27. Demographic data for comparisons between severe malaria subtypes and controls with a history of CM. * denotes mean (standard deviation). Statistical significance assessed using a two-tailed t-test.

A. CM versus controls with a history of CM

	Cases	Controls	p value
n	8	8	
Female	1	1	
Age* (yrs)	3.37 (0.92)	3.50 (0.93)	0.68
Parasitemia* (parasite/μL)	189,211 (188,830)	37,837 (56,511)	0.10
BCS			
5	0	8	
2	5	0	
1	3	0	
Hgb* (g/dL)	7.90 (1.80)	9.55 (1.43)	0.10

B. SMA versus controls with a history of CM

	Cases	Controls	p value
n	6	6	
Female	5	5	
Age* (yrs)	3.00 (1.26)	3.00 (1.26)	1
Parasitemia* (parasite/μL)	66800 (66173)	60200 (86803)	0.78
BCS			
5	4	6	
4	1	0	
3	1	0	
Hgb* (g/dL)	3.35 (1.00)	9.45 (1.43)	5.27E-05

C. Concurrent CM and SMA versus controls with a history of CM

	Cases	Controls	p value
n	4	4	
Female	2	2	
Age* (yrs)	3.75 (0.96)	3.75 (0.96)	1
Parasitemia* (parasite/μL)	19287 (34975)	3962 (6010)	0.37
BCS			
5	0	4	
2	1	0	
1	3	0	
Hgb* (g/dL)	2.97 (0.75)	8.77 (2.27)	0.023

Supplemental Table 28. Top 20 transcripts with the most significant association from differential gene expression analyses between CM in comparison to controls with a history of CM. Significance of association was assessed with a quasi-likelihood F test based on the negative binomial distribution. False discovery rate was used to correct for multiple testing.

Gene	logFC	logCPM	p value	FDR
IL1R2	4.13	9.07	2.15E-09	1.51E-05
CD163	3.36	5.35	7.86E-08	2.77E-04
FKBP5	2.18	7.86	3.55E-07	8.34E-04
MCEMP1	2.75	6.32	5.04E-07	8.87E-04
SAMSN1	2.60	6.66	1.07E-06	1.51E-03
SOCS3	2.07	6.75	2.01E-06	2.23E-03
TRABD2A	-1.82	7.02	2.22E-06	2.23E-03
KCNE1	2.63	7.61	5.58E-06	4.39E-03
SPATC1	2.97	4.79	5.61E-06	4.39E-03
IL10	3.49	4.45	8.68E-06	6.12E-03
ETS2	1.91	5.90	1.16E-05	7.09E-03
AATBC	-2.97	4.63	1.30E-05	7.09E-03
MMP8	3.66	6.04	1.31E-05	7.09E-03
TRIB1	2.78	5.06	2.14E-05	0.010
CCR5AS	3.57	4.52	2.22E-05	0.010
DAAM2	4.50	3.80	3.30E-05	0.014
S100A12	2.31	9.91	3.35E-05	0.014
BMX	2.76	4.52	3.90E-05	0.015
PFKFB3	1.76	7.54	4.58E-05	0.016
OLFM4	4.09	4.20	4.58E-05	0.016

Supplemental Table 29. Top 20 transcripts with the most significant association from differential gene expression analyses between concurrent CM and SMA in comparison to controls with a history of CM. Significance of association was assessed with a quasi-likelihood F test based on the negative binomial distribution. False discovery rate was used to correct for multiple testing.

Gene	logFC	logCPM	p value	FDR
MPO	8.77	7.40	9.80E-09	3.52E-05
IFI44L	-6.49	6.08	9.61E-08	1.57E-04
ELANE	9.93	6.41	1.31E-07	1.57E-04
ISG15	-4.34	7.30	4.37E-07	3.92E-04
EDIL3	8.74	6.10	6.81E-07	4.89E-04
IFIT1	-5.32	5.80	9.02E-07	5.40E-04
ZDHHC19	4.68	5.65	1.11E-06	5.71E-04
OAS3	-3.71	6.23	1.57E-06	7.04E-04
IL1R2	4.34	8.21	2.18E-06	8.70E-04
PRTN3	8.95	5.51	2.53E-06	9.07E-04
CTSG	8.94	5.51	4.01E-06	1.31E-03
MMP9	4.10	8.49	4.63E-06	1.38E-03
RBFOX1	6.66	5.72	5.36E-06	1.48E-03
IFIT3	-3.39	7.10	6.49E-06	1.66E-03
MMP8	5.40	5.11	1.07E-05	2.56E-03
OLFM4	4.57	5.73	1.21E-05	2.71E-03
BPI	4.60	5.87	1.37E-05	2.76E-03
ARG1	3.48	5.94	1.43E-05	2.76E-03
XAF1	-3.16	6.87	1.46E-05	2.76E-03
CD163	5.55	4.99	1.59E-05	2.86E-03

Supplemental Table 30. Statistical associations between deconvolution based cell proportions and case control status using a two-tailed t test.

Cell type	CM v controls wo history	SMA v controls wo history	Concurrent v controls wo history	CM v controls w history	SMA v controls w history	Concurrent v controls w history
B cells	0.53	0.53	0.56	0.77	0.99	0.44
T cells	0.54	0.051	0.064	0.11	0.0055	0.16
Neutrophils	0.4	0.44	0.71	0.12	0.22	0.23

Supplemental Table 31. Top 20 transcripts with the most significant association from differential gene expression analyses comparing CM versus controls with uncomplicated disease without a history of CM. Analyses are adjusted for B cell, T cell, and neutrophil proportions. Significance of association was assessed with a quasi-likelihood F test based on the negative binomial distribution. False discovery rate was used to correct for multiple testing.

Gene	logFC	logCPM	p value	FDR
IL1R2	3.97	8.73	3.47E-17	2.92E-13
FKBP5	2.30	7.73	2.19E-11	9.19E-08
CD163	4.05	5.50	3.47E-09	9.73E-06
IL18R1	1.98	6.35	1.16E-08	2.31E-05
KCNE1	1.94	7.43	1.37E-08	2.31E-05
MMP8	3.45	5.11	1.78E-08	2.49E-05
MMP9	1.95	7.79	1.30E-07	1.53E-04
OLFM4	4.72	4.33	1.46E-07	1.53E-04
LTF	2.90	4.84	3.00E-07	2.69E-04
RBM33	-1.43	8.29	3.20E-07	2.69E-04
IL1R1	1.81	5.81	8.02E-07	6.13E-04
ADARB1	-1.30	7.17	9.19E-07	6.44E-04
SPATC1	4.98	4.51	1.13E-06	7.32E-04
DAAM2	3.73	4.06	1.79E-06	1.07E-03
KMT2D	-1.60	8.81	2.68E-06	1.50E-03
RORC	-2.45	5.22	3.08E-06	1.60E-03
IGHV3-23	2.04	5.67	3.24E-06	1.60E-03
IRAK3	1.26	8.01	3.56E-06	1.62E-03
PPM1F	-1.22	7.19	3.65E-06	1.62E-03
ZDHHC20	1.59	6.02	4.55E-06	1.91E-03

Supplemental Table 32. Transcripts with significant association from differential gene expression analyses comparing SMA versus controls with uncomplicated disease without a history of CM. Analyses are adjusted for B cell, T cell, and neutrophil proportions. Significance of association was assessed with a quasi-likelihood F test based on the negative binomial distribution. False discovery rate was used to correct for multiple testing.

Gene	logFC	logCPM	p value	FDR
SLC6A19	21.44	7.67	4.05E-07	3.33E-03
LTF	6.12	6.59	1.05E-06	4.33E-03
LCN2	7.15	5.62	5.43E-06	0.015
ARG1	3.23	6.24	7.41E-06	0.015
ELANE	4.38	4.77	1.90E-05	0.031
DEFA1	8.46	4.58	2.96E-05	0.035
DAAM2	6.77	4.35	3.48E-05	0.035
TUBB2A	-18.52	4.40	3.75E-05	0.035
THSD4	4.08	5.31	3.84E-05	0.035

Supplemental Table 33. Top 20 transcripts with the most significant association from differential gene expression analyses comparing concurrent CM and SMA versus controls with uncomplicated disease without a history of CM. Analyses are adjusted for B cell, T cell, and neutrophil proportions. Significance of association was assessed with a quasi-likelihood F test based on the negative binomial distribution. False discovery rate was used to correct for multiple testing.

Gene	logFC	logCPM	p value	FDR
PRKG1	11.01	6.00	3.35E-07	1.30E-03
LY6E	-3.27	7.54	4.14E-07	1.30E-03
HSPA1A	3.09	7.40	4.49E-06	9.39E-03
ATP8B4	5.61	4.89	1.41E-05	0.019
OLFM4	5.23	5.78	1.62E-05	0.019
ISG15	-3.52	5.95	1.79E-05	0.019
OAS3	-3.19	6.39	2.20E-05	0.020
KMT2D	-2.86	9.17	2.84E-05	0.022
RBFOX1	10.25	5.46	3.17E-05	0.022
ARID5B	2.59	6.42	3.90E-05	0.024
ELANE	4.41	6.42	4.81E-05	0.027
FKBP5	2.45	7.65	5.25E-05	0.027
OAS1	-2.31	7.09	5.62E-05	0.027
FRMPD3	-7.51	5.84	6.51E-05	0.029
DPP10	10.03	4.39	8.47E-05	0.035
XAF1	-2.38	7.44	9.96E-05	0.039
RPL21	-2.70	8.88	1.07E-04	0.040
C4B	-10.73	4.67	1.16E-04	0.040
STAP1	-3.71	6.01	1.38E-04	0.046
PRTN3	8.35	5.52	1.48E-04	0.046

Supplemental Table 34. Transcripts with significant association from differential gene expression analyses comparing severe malaria subtypes versus controls with uncomplicated disease with a history of CM. Analyses are adjusted for B cell, T cell, and neutrophil proportions. Significance of association was assessed with a quasi-likelihood F test based on the negative binomial distribution. False discovery rate was used to correct for multiple testing.

A. Gene associated with cerebral malaria in comparison to controls with a history of CM

Gene	logFC	logCPM	p value	FDR
DEFA4	8.07	5.49	1.82E-06	0.013

B. Top 20 genes associated with concurrent cerebral malaria and severe malarial anemia in comparison to controls with a history of CM

Gene	logFC	logCPM	p value	FDR
SLC2A5	11.38	5.90	3.11E-10	1.12E-06
MPO	9.13	7.42	5.06E-09	9.08E-06
IL1R2	4.99	8.21	1.48E-07	1.77E-04
IFI44L	-6.24	6.10	5.48E-07	4.91E-04
MMP9	4.47	8.49	9.68E-07	5.91E-04
LTF	5.78	6.68	9.88E-07	5.91E-04
EFTUD2	4.62	7.19	2.24E-06	1.15E-03
DEFA4	4.89	8.34	2.94E-06	1.32E-03
IFIT1	-5.15	5.78	4.03E-06	1.61E-03
ISG15	-3.90	7.29	5.14E-06	1.84E-03
OLFM4	6.30	5.76	6.09E-06	1.99E-03
ZDHHC19	4.99	5.65	7.41E-06	2.22E-03
ELANE	11.10	6.44	8.23E-06	2.27E-03
BPI	5.35	5.90	1.42E-05	3.65E-03
OAS3	-3.54	6.22	1.83E-05	4.19E-03
IGHV3-23	4.54	5.65	1.87E-05	4.19E-03
HMGB2	3.32	8.38	2.25E-05	4.74E-03
GOLPH3	3.43	7.30	2.74E-05	5.46E-03
APOL6	-3.66	6.11	4.08E-05	7.01E-03
ANP32E	4.20	6.41	4.08E-05	7.01E-03

Supplemental Table 35. Probes remaining after quality control

Comparison	Number of probes passing QC
CM vs Co w/o hx	8403
CM vs Co w/ hx	7047
SMA vs Co w/o hx	8219
SMA vs Co w/ hx	7233
Concurrent vs Co w/o hx	6269
Concurrent vs Co w/ hx	3590
Co w/ hx vs Co w/o hx	7532
CM vs SMA	6373
CM vs Concurrent	6822
SMA vs Concurrent	6155

Supplemental Table 36. Gene Ontology biological processes with the strongest associations when comparing gene expression between malaria subtypes and uncomplicated malaria controls with a history of CM. Statistical significance was assessed using a one-sided hypergeometric test. P values were adjusted for multiple testing using the Benjamini-Hochberg method.

A) Cerebral malaria versus uncomplicated controls with a history of cerebral malaria, data corresponds to Supplemental Figure 4A

Term for biological process	Gene Ratio	Adjusted p value
Response to hydrogen peroxide	8/66	3.39E-05
Cellular response to hydrogen peroxide	7/66	3.39E-05
Cellular response to reactive oxygen species	8/66	3.39E-05
Response to reactive oxygen species	9/66	3.39E-05
Cellular response to oxidative stress	9/66	2.44E-04
Negative regulation of phosphorylation	10/66	2.54E-04
Cellular response to chemical stress	9/66	4.74E-04
Negative regulation of protein phosphorylation	9/66	4.74E-04
Regulation of DNA-binding transcription factor activity	10/66	4.74E-04
Negative regulation of phosphate metabolic process	10/66	4.74E-04

B) Concurrent cerebral malaria and severe malarial anemia versus uncomplicated controls with a history of cerebral malaria, data corresponds to Supplemental Figure 4B

Term for biological process	Gene Ratio	Adjusted p value
Response to virus	17/65	7.04E-12
Defense response to virus	14/65	1.35E-10
Defense response to symbiont	14/65	1.35E-10
Response to type I interferon	8/65	7.77E-09
Regulation of cytokine-mediated signaling pathway	10/65	2.42E-08
Negative regulation of innate immune response	8/65	2.42E-08
Negative regulation of response to biotic stimulus	9/65	2.42E-08
Regulation of response to cytokine stimulus	10/65	3.64E-08
Cellular response to type I interferon	7/65	7.89E-08
Negative regulation of viral genome replication	7/65	1.22E-07

Supplemental Note 1

Sample R code for transcriptomic analyses:

```
setwd("C:/Workingdirectory*/")
Counts <- read.delim("ExpressionData.txt", comment.char="#")
head(Counts)
library(edgeR)
y <- DGEList(counts=Counts[,2:25], genes=Counts[,1])
Group <- factor(c(1,1,2,2,3,3,4,4,5,5,6,6,7,7,8,8,9,9,10,10,11,11,12,12))
Treatment <-
factor(c("Control","Severe","Control","Severe","Control","Severe","Control","Severe","Control","S
evere","Control","Severe","Control","Severe","Control","Severe","Control","Severe","Control","Se
vere","Control","Severe","Control","Severe"))
data.frame(Sample=colnames(y),Group,Treatment)
design <- model.matrix(~Group+Treatment)
rownames(design) <- colnames(y)
design
keep <- filterByExpr(y, design)
table(keep)
keep
y <- y[keep, , keep.lib.sizes=FALSE]
nrow(y)
y <- calcNormFactors(y)
y$samples
plotMDS(y, col=rep(1:2, gen=1:12))
y <- estimateDisp(y, design, robust=TRUE)
fit <- glmQLFit(y, design, robust=TRUE)
plotQLDisp(fit)
qlf <- glmQLFTest(fit)
topTags(qlf)
summary(decideTests(qlf))
plotMD(qlf)
o <- order(qlf$table$PValue)
cpm <- cpm(y)[o[1:405],]
sigdiff <- topTags(qlf, n=405)
library(gplots)
hits<-data.frame(sigdiff)
tophits<-hits$genes
tophits[tophits==""]<-NA
tophitstrimmed<-na.omit(tophits)
row.names(logcpm)<-Counts$GeneID
final<-logcpm[tophitstrimmed, ]
final.z<-t(apply(final, 1, scale))
final20<-head(tophitstrimmed, n=20)
final20top<-logcpm[final20, ]
final20.z<-t(apply(final20top, 1, scale))
final20topm<-data.matrix(final20.z)
heatmap(final20topm)
heatmap.2(final20topm, Colv=NA, scale="none", col=bluered(100), trace="none",
density.info="none", symm=T, symkey=F, symbreaks=T)
```

Supplemental Note 2

Data Access Instructions

Project:

A shared inflammatory signature across severe malaria syndromes in transcriptomic, proteomic, and metabolomic analyses

Metabolomics Data

Data Repository: MassIVE

Dataset ID: MSV000096913

url: **https://massive.ucsd.edu/ProteoSAFe/dataset.jsp?task=ba8df9f0096c4bf6abb946f323f153c1**

Made public: 03/25/2025

Proteomics Data

Data repository: Pride

Dataset ID: PXD058621

url: **https://www.ebi.ac.uk/pride/archive/projects/PXD058621**

Project accession: PXD058621

Release Date: 03/25/2025

Transcriptomic data:

Data repository: GEO

Dataset ID: GSE289197

Reviewer access details: GSE289197

url: **https://www.ncbi.nlm.nih.gov/geo/query/acc.cgi?acc=GSE289197**

Release Date: 03/25/2025
