# Supplementary material for: A shared inflammatory signature across severe malaria syndromes manifested by transcriptomic, proteomic and metabolomic analyses
Source: Nat Commun. 2025 May 18;16:4620. doi: 10.1038/s41467-025-59281-5 (PMC12086225; doi:10.1038/s41467-025-59281-5)
Supplement: Supplementary file 2 — Description of Additional Supplementary Files [file 41467_2025_59281_MOESM2_ESM.pdf]

Description of Additional Supplementary Files

Supplementary Data 1

“CM vs Uncomplicated, no hx” tab – complete list of transcripts from differential gene expression analyses between CM in comparison to controls without a history of CM. Samples were matched for age, sex, and ethnicity. Significance of association was assessed with a quasi-likelihood F test based on the negative binomial distribution. False discovery rate was used to correct for multiple testing.

“SMA vs Uncomplicated, no hx” tab - complete list of transcripts from differential gene expression analyses between SMA in comparison to controls without a history of CM. Samples were matched for age, sex, and ethnicity. Significance of association was assessed with a quasi-likelihood F test based on the negative binomial distribution. False discovery rate was used to correct for multiple testing.

“Conc vs Uncomplicated, no hx” tab - complete list of transcripts from differential gene expression analyses between concurrent CM and SMA in comparison to controls without a history of CM. Samples were matched for age, sex, and ethnicity. Significance of association was assessed with a quasi-likelihood F test based on the negative binomial distribution. False discovery rate was used to correct for multiple testing.

“CM vs Uncomplicated, w hx” tab - complete list of transcripts from differential gene expression analyses between CM in comparison to controls with a history of CM. Samples were matched for age, sex, and ethnicity. Significance of association was assessed with a quasi-likelihood F test based on the negative binomial distribution. False discovery rate was used to correct for multiple testing.

“Conc vs Uncomplicated, w hx” tab - complete list of transcripts from differential gene expression analyses between concurrent CM and SMA in comparison to controls with a history of CM. Samples were matched for age, sex, and ethnicity. Significance of association was assessed with a quasi-likelihood F test based on the negative binomial distribution. False discovery rate was used to correct for multiple testing.

Supplementary Data 2

“CM vs SMA” tab - complete list of transcripts from differential gene expression analyses between CM in comparison to SMA. Samples were unmatched. Significance of association was assessed with a quasi-likelihood F test based on the negative binomial distribution. False discovery rate was used to correct for multiple testing.

Supplementary Data 3

“Proteomics, CM vs Uncompl” tab – comparison of serum protein from CM cases versus controls without a history of CM. Samples were matched for age, sex, and ethnicity. Statistical significance assessed with a two-tailed paired t test.

“Metabolomics, CM vs Uncompl” tab – comparison of serum metabolites in CM cases versus uncomplicated malaria without history of CM. Statistical significance was assessed with a two-tailed paired t test. False discovery rate was used for multiple testing adjustment.
